# Label-free highly sensitive probe detection with novel hierarchical SERS substrates fabricated by nanoindentation and chemical reaction methods

**DOI:** 10.3762/bjnano.10.239

**Published:** 2019-12-13

**Authors:** Jingran Zhang, Tianqi Jia, Yongda Yan, Li Wang, Peng Miao, Yimin Han, Xinming Zhang, Guangfeng Shi, Yanquan Geng, Zhankun Weng, Daniel Laipple, Zuobin Wang

**Affiliations:** 1 Ministry of Education Key Laboratory for Cross-Scale Micro and Nano Manufacturing, Changchun University of Science and Technology, Changchun 130022, P.R. China; 2 The Key Laboratory of Micro-systems and Micro-structures Manufacturing of Ministry of Education, Harbin Institute of Technology, Harbin, Heilongjiang 150080, P.R. China; 3 Institute of Materials Research, Helmholtz-Zentrum Geesthacht, Max-Planck-Strasse 1, Geesthacht, D-21502, Germany; 4 School of Chemistry and Chemical Engineering, Harbin Institute of Technology, Harbin, Heilongjiang 150010, P.R. China; 5 5Department of Gynaecology, No. 3 Subsidiary Hospital, Harbin Medical University, Harbin, Heilongjiang, 150040, P.R. China

**Keywords:** Ag nanoparticles, hierarchical substrates, malachite green molecules, nanoindentation, nanostructures, R6G, SERS

## Abstract

Nanostructures have been widely employed in surface-enhanced Raman scattering (SERS) substrates. Recently, in order to obtain a higher enhancement factor at a lower detection limit, hierarchical structures, including nanostructures and nanoparticles, appear to be viable SERS substrate candidates. Here we describe a novel method integrating the nanoindentation process and chemical redox reaction to machine a hierarchical SERS substrate. The micro/nanostructures are first formed on a Cu(110) plane and then Ag nanoparticles are generated on the structured copper surface. The effect of the indentation process parameters and the corrosion time in the AgNO_3_ solution on the Raman intensities of the SERS substrate with hierarchical structures are experimentally studied. The intensity and distribution of the electric field of single and multiple Ag nanoparticles on the surface of a plane and with multiple micro/nanostructures are studied with COMSOL software. The feasibility of the hierarchical SERS substrate is verified using R6G molecules. Finally, the enhancement factor using malachite green molecules was found to reach 5.089 × 10^9^, which demonstrates that the production method is a simple, reproducible and low-cost method for machining a highly sensitive, hierarchical SERS substrate.

## Introduction

Surface-enhanced Raman scattering (SERS) has triggered significant research interest due to its suitability as an analytical tool for the ultrasensitive detection of molecules [1–4]. Compared with traditional Raman scattering technology, SERS is a surface phenomenon associated with the amplification of Raman intensity by several orders of magnitude for analyte molecules. The high resolution results from the combination of chemical (CM) [5] and electromagnetic enhancement (EM) [6–7]. The CM enhancement is the main factor for charge transfer between the SERS substrate and probe molecule. The EM field enhancement is the main factor for localized surface plasmon resonance (LSPR) and significantly depends on the induced near-field intensity. The size, shape, and interparticle spacing of the nanoparticles or nanostructures influences the LSPR. A series of metals including Au, Cu and Ag are useful in ultra-trace biological or chemical sensing and have shown great potential to obtain surface enhancement. Thus, many different nanoparticles or micro/nanostructures can be applied as a SERS sensor to detect adsorbed markers.

Generally, nanoparticles can be fabricated as SERS substrates at low cost and high production via chemical synthesis methods [8–13], including chemical/electrochemical deposition and electrochemical etching. For instance, Chen et al. [8] employed an electrochemical etching method to fabricate nanocube structures on a Cu_30_Mn_70_ surface by controlling the voltage. In addition, Zhang et al. [10] showed that gold nanoparticles can be fabricated by a gold etchant on a silicon surface as SERS substrates where the optimized enhancement factor was determined to be 8.6 × 10^6^. Zhong et al. [11] presented nanoparticles formed by HAuCl_3_ and sodium citrate solutions on the poly(methyl methacrylate) (PMMA) template as a transparent SERS substrate. Then, malachite green at a concentration of 0.1 nmol/L was detected using the AuNPs/PMMA film SERS substrates. Zhang et al. [12] fabricated core–shell structures comprised of SiO_2_ and gold with a sub-10 nm shell thickness by adding HAuCl_4_ and the reducing agent K_2_CO_3_ on the SiO_2_ surface and found that the SERS enhancement becomes weaker with increasing shell thickness. However, nanoparticles fabricated by the chemical synthesis method have poor reproducibility and uneven density, and the hot spot position is difficult to accurately determine.

Recently, many nanostructure shapes have been fabricated for use as SERS substrates including nanorods [14–16], nanostars [17–18], nanoantennas [19], and nanospheres [20]; these have been successfully machined by existing lithography-based technologies. Furthermore, hierarchical substrates have also been fabricated by a combination of lithography [21–25] and self-assembly. Matricardi et al. [21] used the template-assisted assembly of gold nanospheres with patterned PDMS molds featuring square array geometries with lattice parameters of 400 to 1600 nm and hole diameters of 230 to 960 nm. Then 4-acetamidothiophenol at 10^−4^ mol/L was detected using this substrate. Domenici et al. [22] used electron beam lithography and self-assembly methods to fabricate gold clusters of micrometer size and regular spacing. Subsequently, the detection resolution of 4-acetamidothiophenol was 0.05 g/L using the substrate. Nanoparticle cluster array structures with a size of 40 nm were fabricated by electron beam lithography and self-assembly methods [23]. *Bacillus cereus* and *Staphylococcus aureus* were detected with this substrate. However, the major limitation of lithography-based methods is the difficulty in machining more complex nanostructures, particularly complex 3D structures.

Recently, tip-based micro/nanomachining methods [26–30] have been used to fabricate SERS substrates with two main advantages. First, the wear of the tip is negligible during machining on the metal surface due to the low hardness of the metals used (copper, aluminum). Second, the micro/nanomechanical machining method is ideal for the fabrication of micro/nanostructures because it allows for control of normal force or displacement and thus the depth of the micro/nanostructures is accurately achieved. Furthermore, the micro/nanomechanical machining method is suitable for machining more complex micro/nanostructures. Therefore, arrayed microcavities could be machined by a tip-based indention method previously described by us [31]. This prior work [31] is mainly concerned with the fabrication of arrayed inverted pyramid cavities as SERS substrates using an indentation method that studied the effect of the Raman intensity of R6G molecules on the arrayed inverted pyramid cavities of Cu(110) substrates with different feeds. In the present work, a new method including an indentation process and chemical redox reaction is achieved to machine the hierarchical SERS substrates. Complex arrayed micro/nanocavities are formed on the Cu(110) plane by changing the period of the force signal and the machining feed. Ag nanoparticles were generated via redox on the cavities and pile-up of the Cu(110) surface. Different structures of silver nanoparticles and copper surfaces can be produced by changing the corrosion time, and R6G molecules are used as adsorption markers. Finally, the Raman intensities of malachite green molecules with low concentration are detected on the optimized hierarchical SERS substrates. This work combines force modulation indentation with chemical reaction methods. The hierarchical substrates include not only complex micro/nanostructures, but also Ag nanoparticles are generated on the Cu(110) surface. This method can be successfully applied to discriminate pesticide residues or viruses at very small quantities.

## Results and Discussion

### Morphological characterization for various feeds

**Figure 1:**
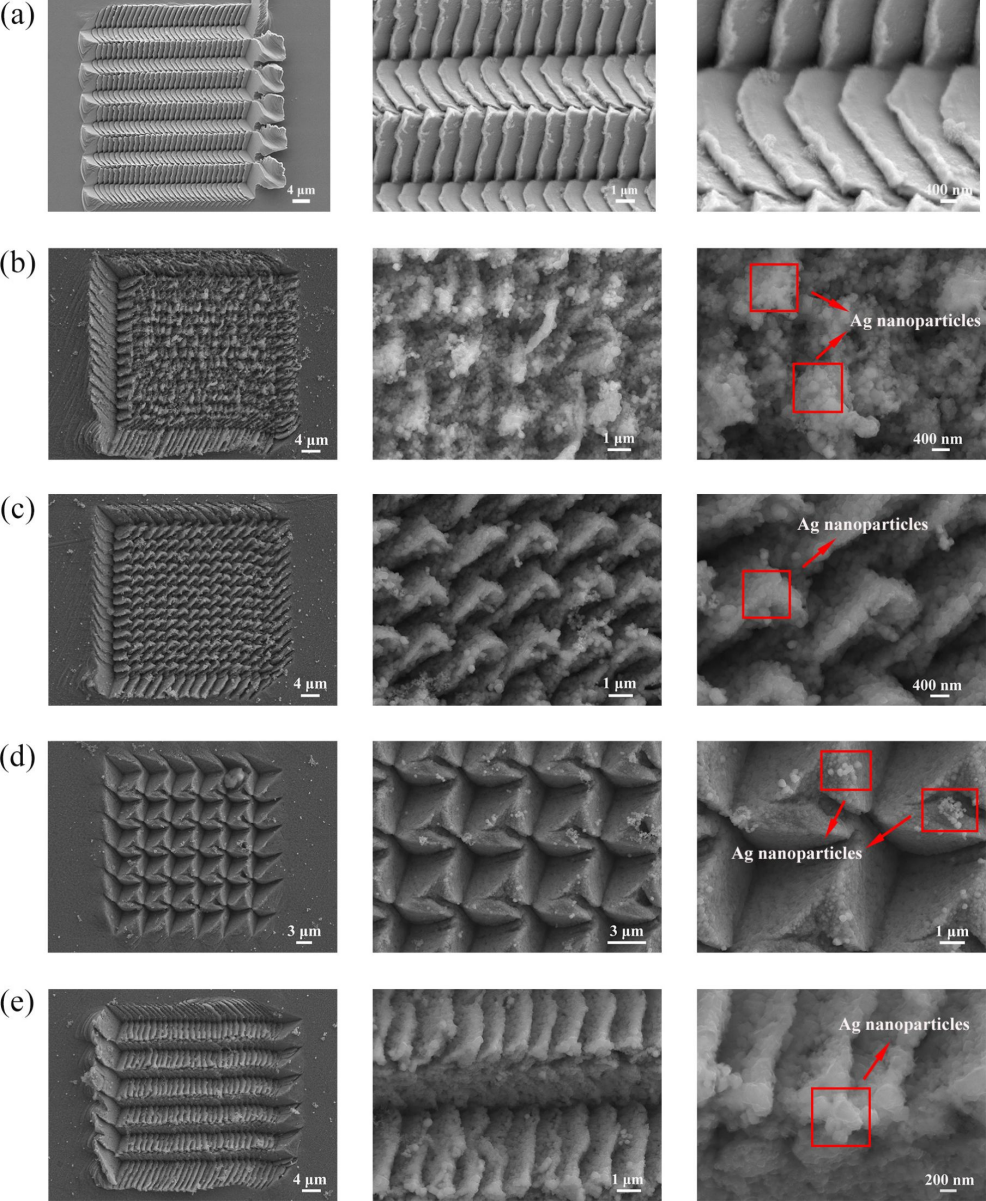
SEM images of the arrayed triangular cavities with different feeds in AgNO_3_ solution before and after 5 minutes: (a) *f_x_* = 5 μm and *f_y_* = 1 μm before using AgNO_3_ solution, (b) *f_x_* = 2 μm and *f_y_* = 1 μm after using AgNO_3_ solution, (c) *f_x_* = 2 μm and *f_y_* = 2 μm, (d) *f_x_* = 5 μm and *f_y_* = 5 μm, (e) *f_x_* = 5 μm and *f_y_* = 1 μm.

Scanning electron microscopy (SEM) images of the arrayed triangular cavities fabricated by different feeds before and after five minutes of corrosion time in AgNO_3_ solution are shown in Figure 1. Figure 1a shows SEM images of arrayed triangular cavities before corrosion of HCl and AgNO_3_. The triangular cavities form a structure similar to fish scales with *f_x_* = 5 μm and *f_y_* = 1 μm. Additionally, the inside of the cavities and the surface of the sample are smooth. However, the surface of the sample, the inside of the cavities, and pile-ups of material are roughened when using HCl and AgNO_3_ solutions. Ag nanoparticles are generated on different positions including inside the cavities, in the pile-ups of material, and the surface of the sample as shown in Figure 1b–e. Figure 1b shows SEM images of arrayed triangular cavities etched by AgNO_3_ with *f_x_* = 2 μm and *f_y_* = 1 μm. More complex structures can be generated via the adjacent cavities that are overlapped and squeezed using the normal force control method with a decrease in feed (*f*_y_).

The nanoparticles are generated inside of the cavities and the pile-ups of the cavities using the AgNO_3_ solution. The SEM images of arrayed pyramidal cavities etched by AgNO_3_ solution are formed with *f_x_* = 2 μm and *f_y_* = 2 μm, as shown in Figure 1c. The smaller pyramid cavities are formed via a feed of 2 μm in the two directions. More Ag nanoparticles are generated on the smaller pyramid cavities. The adjacent cavities are just overlapped with *f_x_* = 5 μm and *f_y_* = 5 μm, as shown in Figure 1d. Compared with the inside of the cavities, more Ag nanoparticles are generated in the pile-up regions of the cavities. The fish-scale-like pattern induced by machining is formed with *f_x_* = 5 μm and *f_y_* = 1 μm, as shown in Figure 1e. Compared to the original arrayed pyramid cavities, the structures of different positions are roughed using AgNO_3_ solution. In addition, 200 nm nanoparticles are formed in the different positions of cavities as shown below in Figure 1e.

**Figure 2:**
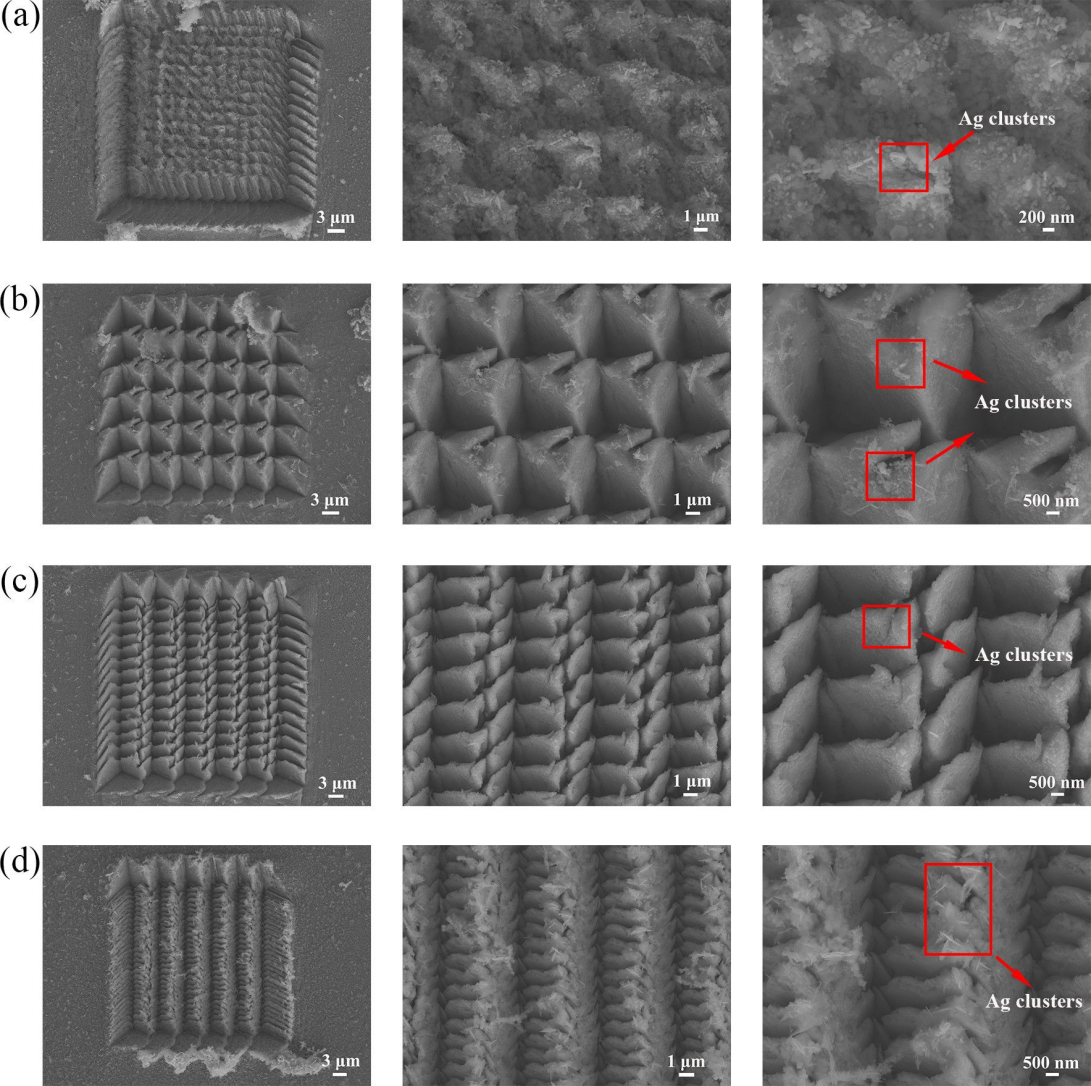
SEM images of the arrayed triangular cavities with different feeds in AgNO_3_ solution for 10 minutes: (a) *f_x_* = 2 μm, *f_y_* = 2 μm, (b) *f_x_* = 5 μm, *f_y_* = 5 μm, (c) *f_x_* = 5 μm, *f_y_* = 2 μm, and (d) *f_x_* = 5 μm, *f_y_* = 1 μm.

Figure 2 shows the SEM images of the arrayed triangular cavities machined by different feeds after a corrosion time of ten minutes in AgNO_3_ solution. When the etching time is increased, the clustering structures are gradually generated due to the increased dimension of Ag nanoparticles. Figure 2a shows SEM images of arrayed triangular cavities with *f_x_* = 2 μm and *f_y_* = 2 μm at an etching time of 10 minutes in AgNO_3_ solution. Compared with the pyramidal cavities etched for 5 minutes, the topography of the pyramidal cavities etched by 10 minutes are obviously changed as shown in Figure 2c. Figure 1e and Figure 2d show SEM images of arrayed triangular cavities with *f_x_* = 5 μm and *f_y_* = 1 μm. Compared with cavities etched for 5 minutes, more nanoparticles are generated in the cavities and the pile-up of cavities, and the clustering structures are formed in the pile-up regions of the cavities. A similar situation occurs with a feed of 5 μm in the two directions. The nanoparticles etched for 5 minutes are only formed in the pile-up regions of cavities as shown in Figure 1d.

### Elemental analysis of the arrayed pyramidal cavities

The parameters of the feeds (*f_x_, f_y_*) for fabricating micro/nanostructures using the method presented are shown in Table 1.

**Table 1:** Parameters of the feeds in the *x*- and *y*-direction for fabricating micro/nanostructures on the Cu(110) surface.

	(1)	(2)	(3)	(4)	(5)	(6)	(7)	(8)	(9)	(10)

*f_x_* (μm)	10	5	5	5	5	5	2	2	2	2
*f_y_* (μm)	10	5	4	3	2	1	4	3	2	1

**Figure 3:**
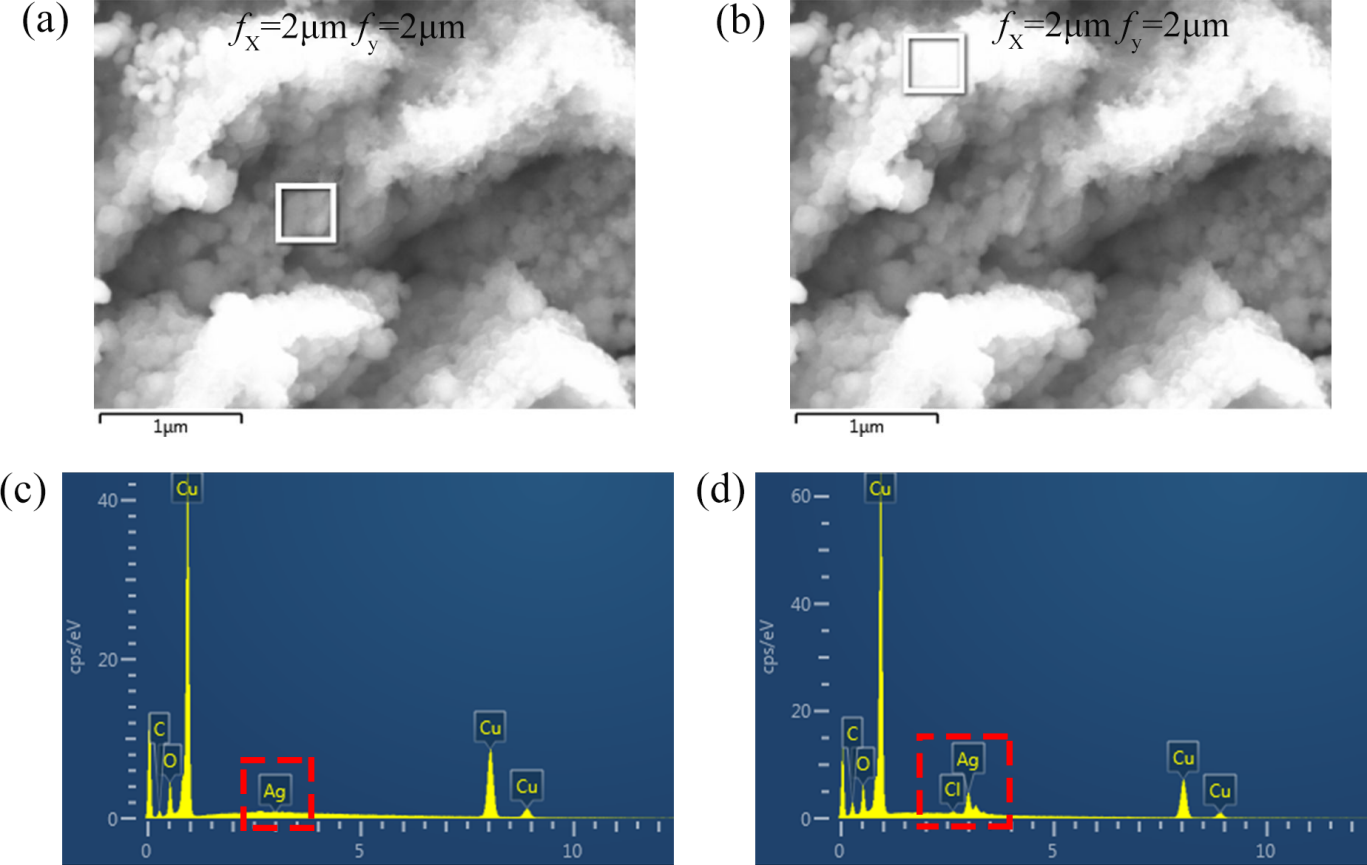
(a) SEM image of the arrayed triangular cavities with *f_x_* = 2 μm, *f_y_* = 2 μm in AgNO_3_ solution after 5 minutes. (b) SEM image of the pile-ups of pyramidal cavities with *f_x_* = 2 μm, *f_y_* = 2 μm in AgNO_3_ solution after 5 minutes. (c) EDX image of the arrayed triangular cavities with *f_x_* = 2 μm, *f_y_* = 2 μm. (d) EDX image of the pile-ups of the pyramidal cavities with *f_x_* = 2 μm, *f_y_* = 2 μm.

Figure 3 shows SEM and energy-dispersive X-ray spectroscopy (EDX) images of the arrayed triangular cavities with *f_x_* = 2 μm, *f_y_* = 2 μm in AgNO_3_ solution for 5 minutes. The nanoparticles are generated on the Cu(110) plane as shown in Figure 3a,b. The elements of the hierarchical surface contain copper, silver, and oxygen as shown in Figure 3c,d. Copper is the main element in the substrate, and Ag is formed on the Cu substrate due to redox reactions. Oxygen is generated when the Ag nanoparticles are formed. Table 2 and Table 3 show the distribution of the contents of each element of the internal cavities and pile-ups of cavities with *f_x_* = 2 μm and *f_y_* = 2 μm. The mass ratio of silver is 10.25% on the pile-ups of cavities. However, the mass ratio of silver is only 0.64% on the inside of the cavities. The atomic ratio of silver is 4.12% and 0.25% on the pile-ups of the cavities and inside of the cavities, respectively. This shows that the content of silver on the pile-ups of the cavity is higher than the content of silver on the inside of the cavities.

**Table 2:** The elemental content of the internal cavities for the hierarchical SERS substrate with the machining feed of *f_x_* = 2 μm and *f_y_* = 2 μm.

Element	Weight percent	Atomic percent

C	6.57	23.07
O	7.63	20.12
Cu	85.16	56.56
Ag	0.64	0.25
total	100	100

**Table 3:** The elemental content of the pile-ups of the cavities for the hierarchical SERS substrate with a machining feed of *f_x_* = 2 μm and *f_y_* = 2 μm.

Element	Weight percent	Atomic percent

C	7.36	23.07
O	6.41	20.12
Cl	0.18	0.22
Cu	75.79	51.71
Ag	10.25	4.12
total	100	100

**Figure 4:**
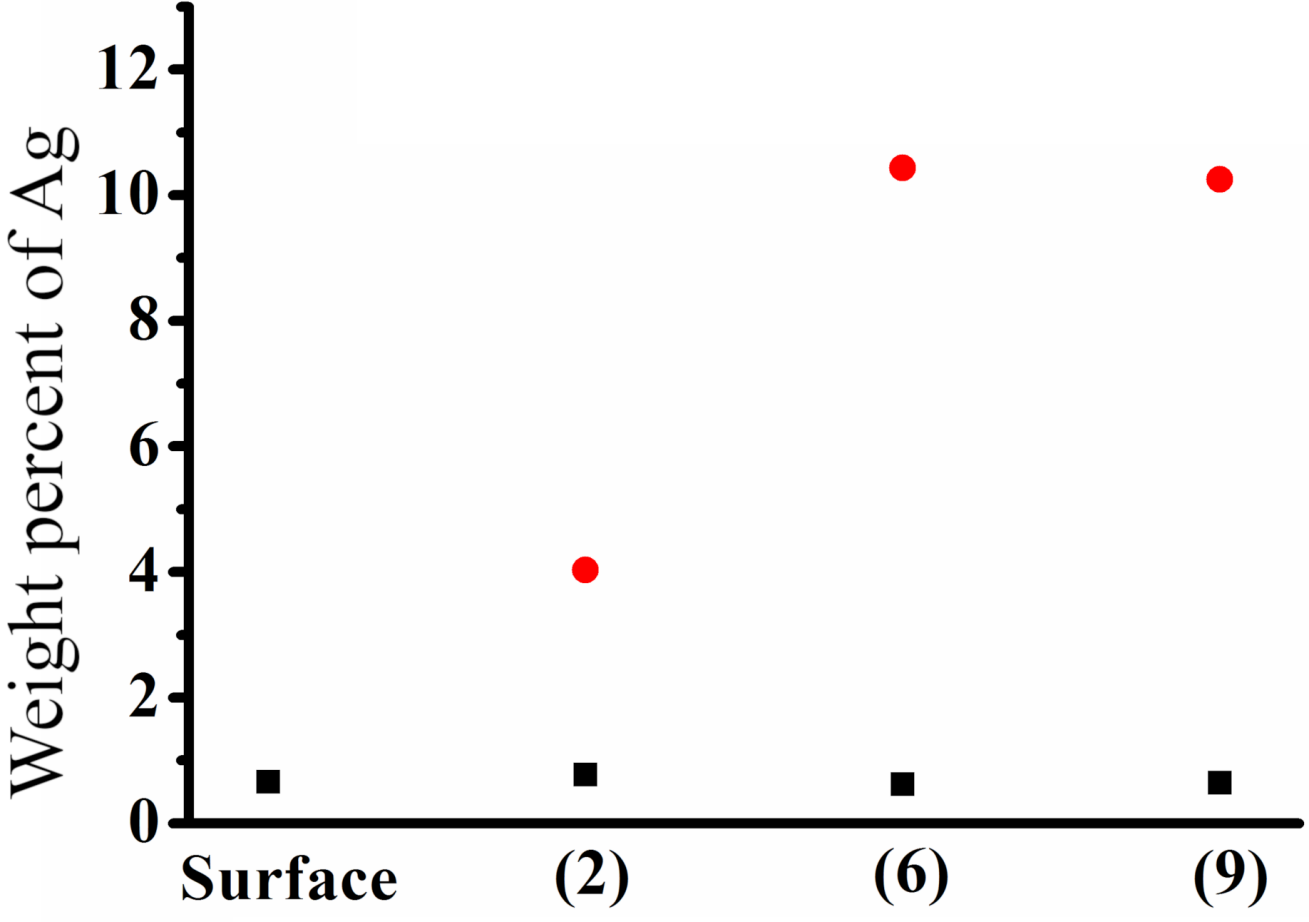
Weight percent of Ag for structures prepared with different feeds in the internal cavity (black squares) and the pile-ups of cavities (red circles). The details of the feed structures are given in Table 1.

Figure 4 shows weight percent of Ag with the structures of different feeds in the internal cavity and the pile-ups of the cavities and the content of each element in the internal cavities and the pile-ups of the cavities with different feeds. Similar conclusions are obtained for different arrayed pyramidal cavities with different feeds. The amount of Ag nanoparticles with pile-ups of cavities is higher than the amount in the internal cavities as shown in Figure 4. Compared with other structures prepared with different feeds, such as *f_x_* = 5 μm, *f_y_* = 1 μm or *f_x_* = 2 μm, *f_y_* = 2 μm, the amount of Ag nanoparticles is lower in the pile-ups of the cavities with *f_x_* = 5 μm and *f_y_* = 5 μm.

Arrayed pyramids were fabricated with different feeds and showed that the Ag nanoparticles are formed on the internal cavities, while the pile-ups of cavities contain much higher Ag nanoparticle content formed on the plane during chemical corrosion. The stress in the cavity and in the plane are different due to the pile-up of material generated during the fabrication of arrayed pyramidal cavities. The change in stress on the different positions has a significant effect on chemical corrosion. The deformation of the material and the defection of the structure are due to the generation of stress during the machining of the arrayed pyramidal cavities.

The replacement reaction is achieved in the single crystal copper surface and the AgNO_3_ solution and Ag nanoparticles are formed on the different positions of the single crystal copper surface. The single crystal copper surface becomes rougher and more defects are formed after the etching process. Furthermore, the defects of the internal cavities and the pile-ups are much more than on the surface of single crystal copper. Compared with the single crystal copper surface, the internal cavities and pile-ups of cavities are more easily etched by AgNO_3_ solution. Therefore, the stress state affects the corrosion structure. Compared with the single crystal copper surface, more silver nanoparticles are formed in the internal cavities and pile-ups of cavities.

### Raman spectroscopy of the hierarchical Ag/Cu substrates

#### Microstructure optimization with different machining parameters

The AgNO_3_ solution corrosion time and different machining parameters have a significant influence on the performance of the hierarchical SERS substrates. R6G is employed as the Raman probe to quantify the Raman enhancement performance. Thus, the Raman spectra are obtained for hierarchical SERS substrates with different machining parameters and corrosion time.

**Figure 5:**
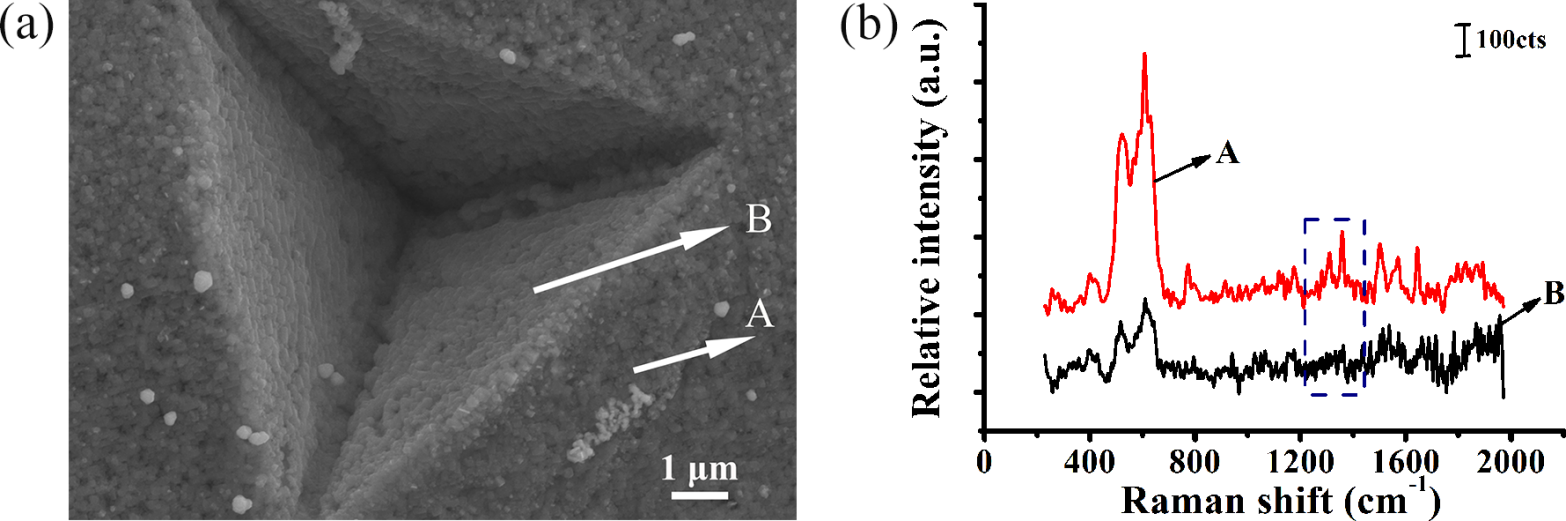
(a) SEM image of a single Ag/Cu pyramidal structure on the substrate with a feed of *f*_x_ = 10 μm, *f*_y_ = 10 μm. (b) Raman spectrum of R6G molecules at 10^−8^ mol/L on a single Ag/Cu substrate, where A and B represent the pile-ups and internal area of the cavity, respectively.

Figure 5a shows a SEM image of a single cavity on the Ag/Cu substrate with *f*_x_ = 10 μm and *f*_y_ = 10 μm and Figure 5b shows the Raman spectrum of R6G molecules at 10^−8^ mol/L on the single Ag/Cu substrate where spectra A and B correspond to the pile-ups of the cavities and the internal cavity, respectively. Figure 5b illustrates stronger enhancement in the pile-ups of the cavity versus in the internal cavity.

**Figure 6:**
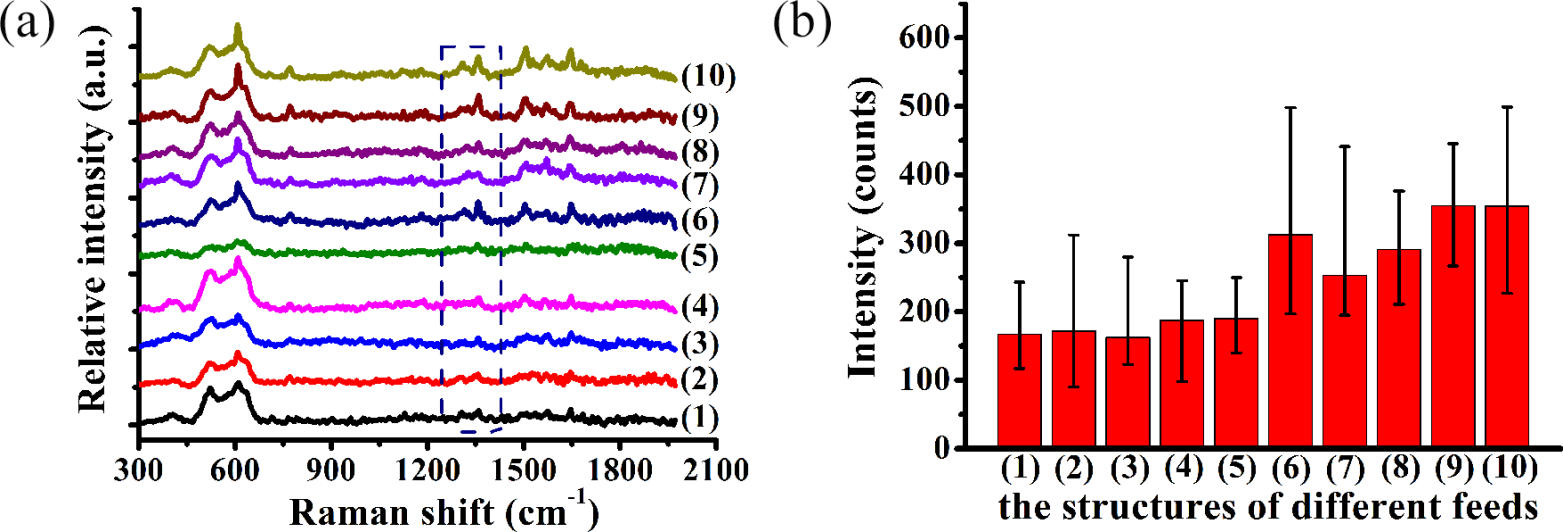
(a) Raman spectrum of R6G molecules at 10^−8^ mol/L with different feeds, as defined in Table 1, on Ag/Cu substrates with a corrosion time of 5 minutes. (b) Average Raman intensity of the 1362 cm^−1^ peak for the structured Ag/Cu substrates with different feeds.

Figure 6 shows the Raman spectrum of R6G molecules at 10^−8^ mol/L for hierarchical SERS substrates with a corrosion time of 5 minutes in AgNO_3_ solution. The Raman spectra peaks of R6G molecules are identified as 611, 772, 1183, 1312, 1362, 1504, and 1604 cm^−1^ on the different hierarchical substrates as shown in Figure 6a. Figure 6b shows the average Raman intensity of R6G of the 1362 cm^−1^ peak of the arrayed triangular cavities with different feeds with a corrosion time of 5 minutes in AgNO_3_ solution (see Figure 6a). The parameters of the feeds (*f_x_, f_y_*) for fabricating micro/nanostructures using this method are shown in Table 1.

When the feed in the two directions ranges from 3–5 μm, the Raman intensities do not change significantly, as shown in Figure 6b. However, compared with other machining parameters, the Raman intensity exhibits a stronger enhancement with three hierarchical structures including those labeled as (6), (9) and (10) as shown in Figure 6b. The “fish scale” structures are formed when the adjacent cavities are overlapped and squeezed. This generates a stronger enhancement for the “fish scale” structures than those in the plane. Compared with the other structures, more Ag nanoparticles are generated on the “fish scale” structures using the two parameters as shown in Figure 1c and Figure 1e. More Ag indicates that more nanoparticles are formed in the pyramidal cavities, and a stronger electric field is generated between the nanoparticles. Therefore, a higher Raman intensity is detected on the “fish scale” structure.

**Figure 7:**
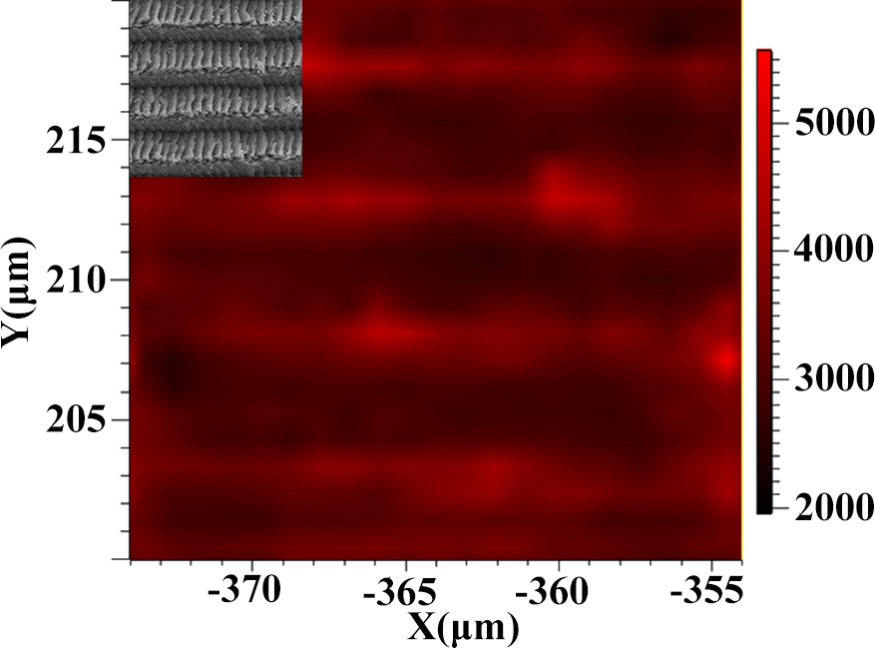
Raman intensity mapping image of the arrayed Ag/Cu substrate with a feed of 5 μm in the *x*-direction and 1 μm in the *y*-direction.

Figure 7 shows a Raman intensity mapping image of the arrayed Ag/Cu substrate with a feed of 5 μm in the *x*-direction and 1 μm in the *y*-direction. The inset is the SEM image corresponding to the ﬁeld map. We experimentally verified that the distribution of Raman intensities is uniform along the same direction. Combining the nanoindentation process method with the chemical redox reaction method led to Ag/Cu substrates with high reproducibility and uniformity.

#### Effect of Raman enhancement performance with different corrosion times

**Figure 8:**
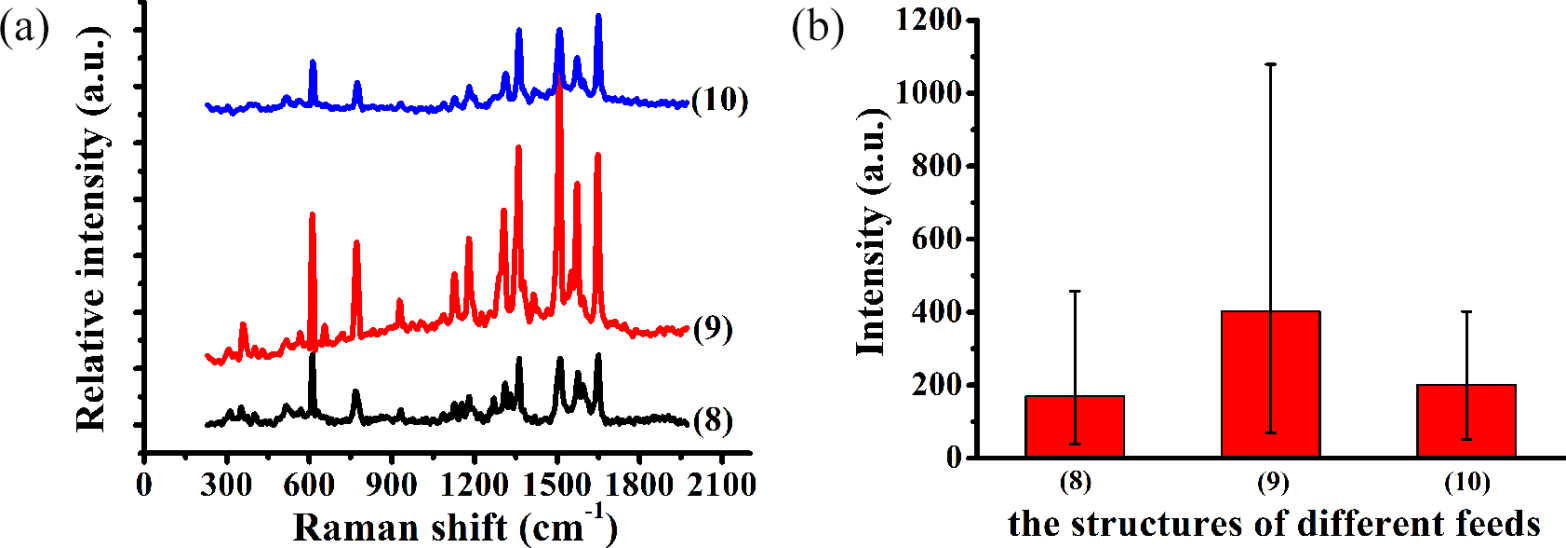
(a) Raman spectra of R6G molecules at 10^−8^mol/L with different feeds on Ag/Cu substrates with a corrosion time of 10 minutes. (b) Average Raman intensity of the 1362 cm^−1^ peak of R6G for the Ag/Cu substrates with different feeds. The details of the structures (8), (9) and (10) are given in Table 1.

In order to research the effect of Raman enhancement using the hierarchical SERS substrate with different corrosion times, the structured single crystal copper was immersed in AgNO_3_ solution for 10 minutes with a concentration of 10^−3^ mol/L. The substrate was then dried using a stream of nitrogen and was soaked in R6G solution of 10^−8^ mol/L for 2 h. The Raman peaks of carbon were generated when a laser power of 2.39 mW (10%) was applied with an etching time of 5 minutes; the Raman signal of R6G molecules was not detected with the etching time of 10 minutes. The R6G molecules are carbonized due to the high Raman intensity. Thus, a significantly lower laser power of 0.016 mW (0.1%) was applied to detect the Raman intensity of the different hierarchical SERS substrates. Figure 8 shows the Raman spectrum of 10^−8^ mol/L R6G on hierarchical SERS substrates with a corrosion time of 10 minutes in AgNO_3_ solution. When the lower laser power was employed, the Raman intensity of R6G molecules could not be detected for structures with other machining parameters. However, the Raman signal of R6G could be detected when *f_x_* = 2 μm and *f_y_* ranges from 1 to 3 μm to as shown in Figure 8a. Compared with the other machining feeds, the Raman signal of R6G is the largest with *f_x_* = 2 μm and *f_y_* = 2 μm. Additionally, the Raman signal for samples with many pile-ups of cavities is ten times larger than the Raman signal within the internal cavities as shown in Figure 8b.

Compared to the internal cavities, the Raman intensity can be enhanced due to more Ag nanoparticles being generated in the pile-ups of cavities. It is indicated that Ag nanoparticles tend to form on the pile-ups of cavities for the hierarchical substrates. However, Ag nanoclusters are formed on the pile-ups of cavities with increase in the corrosion time in AgNO_3_ solution, as shown in Figure 2a,b. The Raman intensity can be enhanced further due to the formation of nanoclusters. Other scholars have already obtained similar conclusions [10,17,32]. Copper nanowires were soaked in AgNO_3_ solution by Wang et al. [10]. A local electric field enhancement is caused when a single nanoparticle comes in contact with the sample and the “hot spots” are formed by multiple nanoparticles to improve the electric field intensity and local electromagnetic field. Zhang et al. [32] deposited a Ag film of 30 nm and a Au film of 10 nm on the roughened silicon surface by the thermal evaporation method. The higher Raman intensity of the sample at 10^−8^ mol/L was detected on the Au@Ag particle substrate. A three-dimensional nanostar structure was fabricated by Gopalakrishnan et al. [17] in a circular groove. The electric field intensity was greatly improved by the adjacent nanostars, and the adenine molecules were detected at low concentration. In previous works, Ag nanoparticles were fabricated by the redox reaction or physical deposition methods. Multiple Ag nanoparticles are formed to further improve the Raman intensity of probe molecules. However, the difference in the conclusions obtained by other scholars is that the Raman intensity at different positions on arrayed pyramidal cavities fabricated by the force-controlled indentation machining method are different, including in the internal cavities and the pile-ups of cavities. Moreover, the Raman signals detected by arrayed pyramidal cavities with different machining feeds are also different.

#### SERS study of the hierarchical structures based on the finite element method

**Figure 9:**
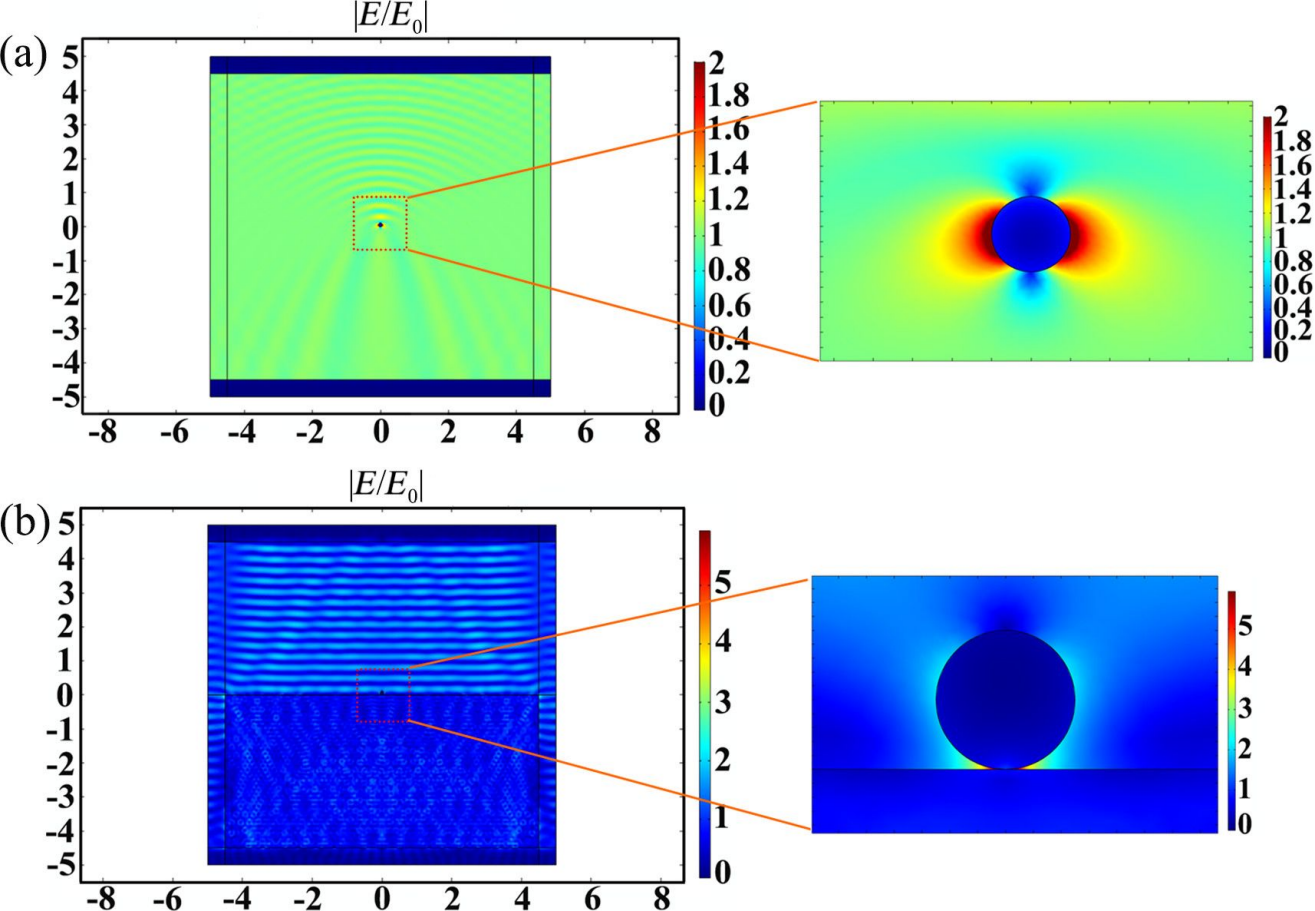
(a) Electric field distribution in the air of a Ag nanoparticle with radius of 100 nm. (b) Electric field distribution of a Ag nanoparticle with radius of 100 nm on the substrate.

We verified the effect of the electric field intensity of the hierarchical structures and modeled the enhancement mechanism of the AgNPs/Cu nanostructures. The local electric field of the AgNPs/Cu nanostructures was calculated using commercial COMSOL software. Figure 9 shows the electric field distribution in the *x*–*z* plane of a single Ag nanoparticle at the air/Cu surface with an incident wavelength of 532 nm. The definitions of the geometrical parameters are provided, where *E*(*x*), *H*(*y*) and *K*(*z*) are the electric field, magnetic field, and direction of light propagation, respectively. The electric field intensity is 2.19 V/m, and the radius of the Ag nanoparticle is 100 nm, as computed in air as shown in Figure 9a. Figure 9b shows the electric field distribution of the Ag nanoparticle with a radius of 100 nm on the Cu substrate. The maximum value of the electric field intensity is mainly concentrated on upon contact between the copper surface and the silver particle, and the electric field intensity is 16.734 V/m. This indicates that the electric field coupling occurs between the copper substrate and the Ag nanoparticle; that is, a new “hot spot” is formed.

**Figure 10:**
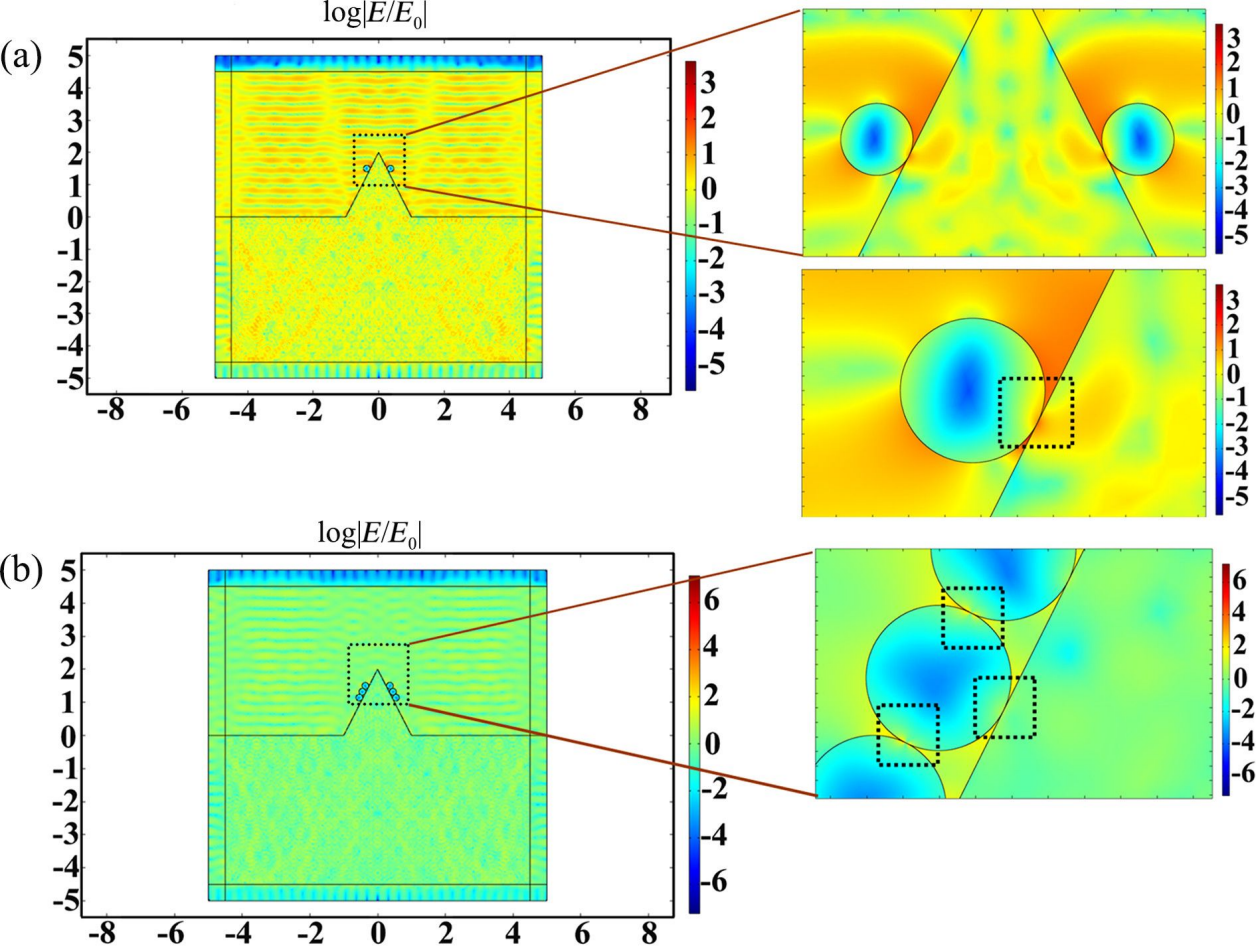
(a) Electric field distribution of a single Ag nanoparticle with a radius of 100 nm on the pile-up of Cu substrate. (b) Electric field distribution of three Ag nanoparticles with a radius of 100 nm on the pile-up of Cu substrate.

Figure 10 shows the electric field distribution of a single and three adjacent silver particles with a radius of 100 nm on a pile-up area of the copper substrate. Figure 10a shows the electric field distribution of the hierarchical structure with a pile-up of the copper structure and a single silver nanoparticle. A hot spot is generated between the pile-up of the copper substrate and the silver particle, and the electric field intensity is 38.899 V/m. The electric field intensity of a single silver particle in contact with the pile-up structure is increased by a factor of about three versus the individual silver nanoparticles on the copper surface. Figure 10b shows the electric field distribution of the hierarchical structures with a pile-up of copper structure and adjacent silver nanoparticle cluster structures where the scale is set to log|*E*/*E*_0_|. A new hot spot is generated due to the interaction with adjacent Ag nanoparticles. At the same time, the other hot spot between the Ag nanoparticle and the pile-up of copper structure is formed and the SERS hot spot intensity is greatly enhanced. The electric field intensity of the three adjacent Ag nanoparticle cluster structures on the pile-up of the Cu substrate is 380 V/m. The electric field intensity is further greatly enhanced compared with a single Ag nanoparticle on the pile-up of the Cu substrate.

The simulation model corresponds to the SEM topography of the indentation structures fabricated by different feeds in the AgNO_3_ solution after 5 minutes as shown in Figure 1d,e. The Ag nanoparticles are formed on the pile-ups of the indentation structures and the adjacent nanoparticles with a radius of 100 nm are contacted with each other, which corresponds well to the simulation model. The simulation results in this section are consistent with the Raman intensities of the R6G molecules detected on the different triangular cavities in the experiment as shown in Figure 6b. As discussed above, the electromagnetic field is generated from the metal nanoparticles. When the AgNPs are on the aggregated copper surface, the electrical field intensity of the plasmonic resonance can be effectively amplified and increased.

The results show that the SERS behavior of the AgNP pyramidal hierarchical substrates can contribute to the following points: (1) the pyramidal cavities and pile-up of copper substrate can be employed as an amplifier and can induce a large electric field intensity. (2) The adjacent AgNPs can generate an additional electrical field enhancement. Theoretically, AgNP pyramidal hierarchical SERS substrates with higher sensitivity can be achieved. Therefore, the combination of Ag nanoparticle clusters on the pile-up of copper surface leads to a new nanogap and improves the density of SERS hotspots. The electric field intensity of the hierarchical substrate on the pile-ups of copper is higher than the hierarchical substrate on the copper plane.

#### Raman spectroscopy of malachite green molecules on hierarchical SERS substrates

Malachite green (MG) is commonly used in food and biological dyes and is one of the most common bactericides in aquaculture. However, malachite green is highly toxic for aquatic animals or mammals and can induce cancer in animals. Thus, MG is significantly detected at low concentrations in water. The maximum concentration of malachite green is less than 2 μg/kg (5.48 × 10^−9^ mol/L) for many national food safety standards. Pesticide residues in food are detected by using many methods including gas chromatography, gas chromatography mass spectrometry, and liquid chromatography. These methods suffer from the need of preprocessing and long analysis time. However, SERS technology is a new option to detect pesticide residues in a fast, simple, and highly sensitive way.

**Figure 11:**
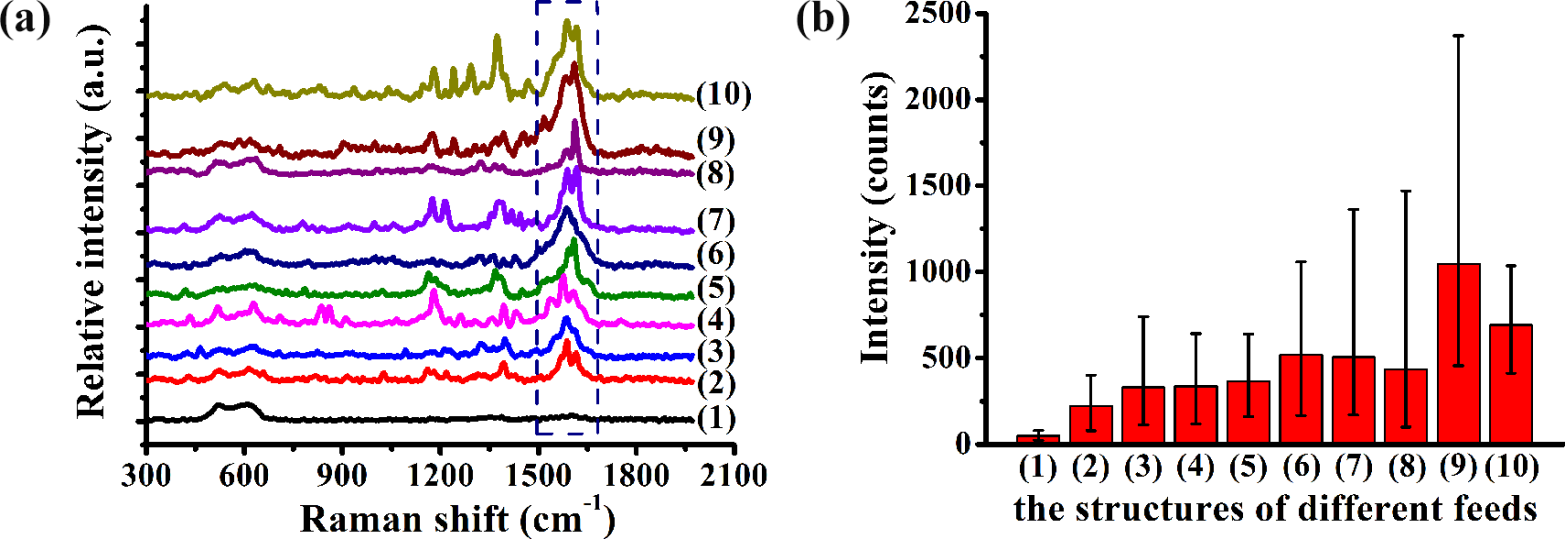
(a) Raman spectra of MG molecules at 10^−7^mol/L on hierarchical SERS Ag/Cu substrates with a corrosion time of 10 minutes with the different feeds. (b) The average Raman intensity of the 1614 cm^−1^ peak of MG molecules in the Ag/Cu structures as a function of different feeds. The feed structures (1)–(10) are described in Table 1.

The characteristic Raman peaks of malachite green molecules were identified at 1172, 1219, 1364, 1394, 1586, and 1614 cm^−1^ on the different hierarchical substrates, as shown in Figure 11.

The incident optical power was 0.148 mW (1%) in the experiment. In this section, the structure of the Ag/Cu substrate was obtained by etching in AgNO_3_ solution for 10 minutes to detect malachite green molecules with a concentration of 10^−7^ mol/L as shown in Figure 11. Figure 11a shows the Raman spectra of the MG molecules for hierarchical SERS substrates with the different feeds. Compared with the other indentation structures, the Raman intensity of MG molecules is weak with separated indentation structures. The Raman intensity of other hierarchical SERS structures gradually increases with decreasing feed.

Figure 11b shows the average Raman intensity of the 1614 cm^−1^ peak of the MG molecules in the Ag/Cu structures with different feeds. The Raman intensity of the MG molecules is 40 ± 10 counts with separated indentation structures. The Raman intensities gradually increase when the feed in the *y*-direction ranges from 1 to 5 μm. Similar to the detection of R6G molecules, the Raman intensities of the three structures are higher than that of structures fabricated by other parameters for the MG molecules including (6), (9), and (10). The “fish scale” structure is formed with *f_x_* = 5 μm, *f_y_* = 1 μm and *f*_x_ = 2 μm, *f*_y_ = 1 μm. The other indentation structures are the formed by the extrusion deformation of the indentation with a feed of 2 μm in the *x*-direction and 2 μm in the *y*-direction, as shown in Figure 3. In particular, the Raman intensity of malachite green is the largest with the feed of 2 μm in the *x*-direction and 2 μm in the *y*-direction. The conclusion regarding the detection of MG molecules is similar to the detection of R6G molecules.

**Figure 12:**
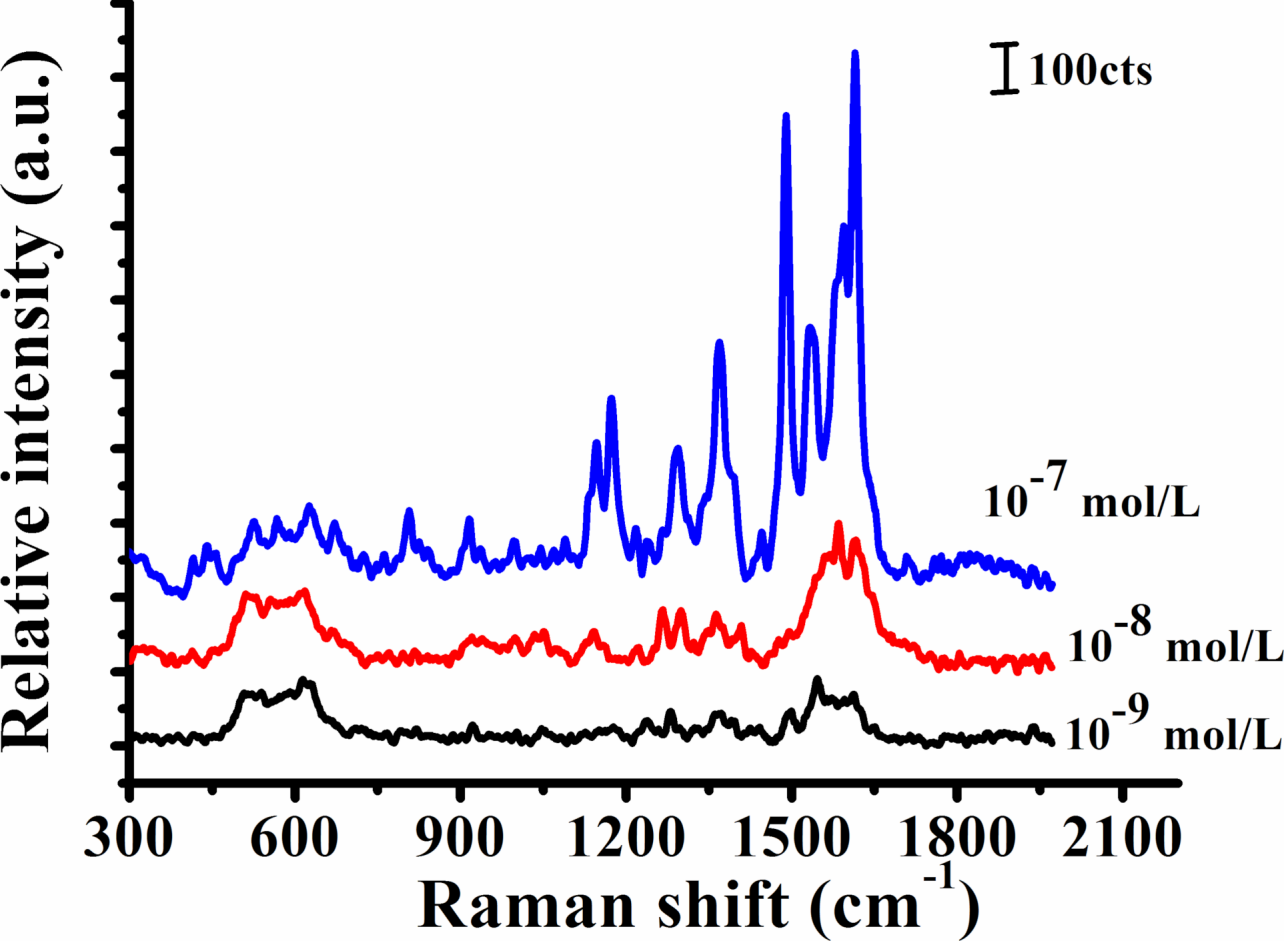
Raman spectrum of MG molecules at different concentrations on Ag/Cu substrates.

The machining parameter is the feed of 2 μm in the *x*-direction and 2 μm in the *y*-direction and the corrosion time is 10 minutes in the AgNO_3_ solution. The detection limit of MG solution is studied for the enhancement performance of hierarchical SERS substrate at 10^−7^ mol/L and 10^−9^ mol/L. The malachite green molecules at 10^−9^ mol/L are detected using the determined optimal processing structure to confirm whether the hierarchical structures can meet known requirements. The detection limit of the hierarchical SERS substrate is 10^−9^ mol/L and meets the national standard (5.48 × 10^−9^ mol/L), as shown in Figure 12.

## Conclusion

We demonstrate a novel method based on indentation and chemical redox reaction to fabricate hierarchical SERS substrates for the detection of probe molecules with high sensitivity. Based on a study of different corrosion times in AgNO_3_ solution, the optimal AgNO_3_ corrosion time was determined to be 10 minutes. The Raman intensity at the pile-ups of materials is higher over the entire energy spectrum as compared to within the internal cavities. This is because the Ag nanoparticles are easily generated on the pile-ups of materials. In addition, the Raman intensity of R6G is higher with a feed of 2 μm in the *x*-direction and a feed of 2 μm in the *y*-direction on the different indentation structures. Third, the intensity and distribution of the electric field of Ag nanoparticles as calculated by Comsol software was shown in the plane substrate and in the cavities. The electric field intensity of Ag nanoparticles on the pile-ups of materials was found to be higher than on the plane substrate. New hot spots are formed at the gap between the adjacent Ag nanoparticles and between Ag nanoparticles and the pile-ups. Finally, the malachite green molecules commonly used in aquatic products are easily detected by the proposed method. This method was shown to be able to meet minimum detection requirements, and the enhancement factor of malachite green molecules is 5.089 × 10^9^ using the optimal indentation structures.

## Experimental

### A novel method to fabricate hierarchical SERS substrates

**Figure 13:**
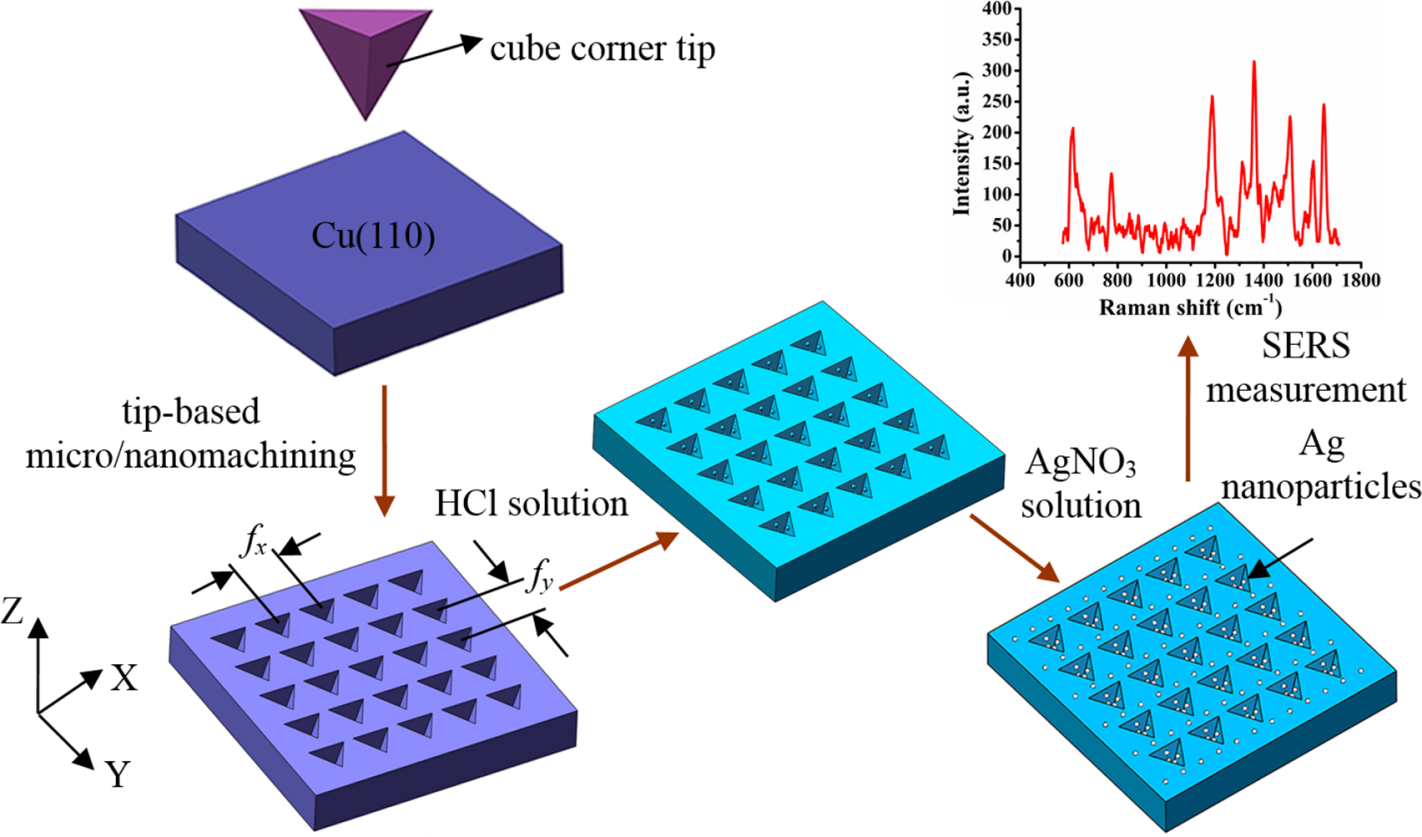
Schematic diagram of the hierarchical SERS substrate with Ag nanoparticles on the arrayed pyramidal cavities.

A single crystal copper (110) plane was used as the sample with dimensions of 5 × 10 × 1 mm. The complex micro/nanostructures are fabricated with different feeds (*f*_x_, *f*_y_) via an indentation method using the cube corner tip with a face angle of 35.26° and a radius of 200 nm. The normal force for the fabrication of the micro/nanostructures is 10 mN, the feed in the *x*-direction ranges from 2 to 10 μm, and the feed in the *y*- direction ranges from 1 to 10 μm. The structured copper surface was cleaned with an excess of alcohol followed by acetone. A 10^−1^ mol/L hydrochloric solution was employed to treat the structured Cu(110) surface for 30 minutes to remove the oxide layer. The concentration of 10^−4^ mol/L AgNO_3_ solution was employed to treat the Cu(110) surface for 5 minutes or 10 minutes. The Ag nanoparticles were generated on the Cu(110) surface, and surface cleaning was carried out further using deionized water to remove any residual AgNO_3_ reagent and copper nitrate production. A stream of nitrogen was then used to dry the hierarchical substrate as shown in Figure 13.

Hydrogen ions play an important role in the surface modification process because they can effectively etch oxide layers, exposing the Cu(110) surface to Ag^+^ at specific areas, as described in Equation 1 and Equation 2. This reaction between Ag^+^ and Cu takes place in the cavities and the Cu(110) surface. This leads to the formation of Ag nanoparticles.

[1] $$\text{CuO}+2\text{HCl}={\text{CuCl}}_{\text{2}}+{\text{H}}_{\text{2}}\text{O}$$

[2] $$\text{Cu}+{\text{AgNO}}_{3}={\text{CuNO}}_{\text{3}}+\text{Ag}$$

In this experiment, EDX/SEM (Zeiss, Germany) was employed to demonstrate that silver was generated on the copper surface. The micro-Raman spectroscopy system (Renishaw, inVia, UK) was equipped with a 532 nm laser and focused with a 50× objective lens. The incident optical power was set to 0.6 mW and the beam diameter was about 1 μm. The signal detector was a Renishaw CCD camera (1040 × 256). The Raman spectrum was validated with a standard Si substrate, without finding specific peaks. The Raman intensity peaks of the R6G probe and malachite green molecules were chosen as 1362 cm^−1^ or 1614 cm^−1^, which are the major Raman peaks for the probe molecules.
